# Physicochemical characteristics, sensory profile, probiotic, and starter culture viability of synbiotic yogurt

**DOI:** 10.5455/javar.2022.i638

**Published:** 2022-12-31

**Authors:** Sadia Jaman, Md. Zakirul Islam, Md. Shahriar Islam Sojib, Md. Sayed Hasan, Md. Mehedi Hasan Khandakar, Md. Sadakatul Bari, Md. Abid Hasan Sarker, Raihan Habib, Mohammad Shohel Rana Siddiki, Mohammad Ashiqul Islam, Md. Harun-ur-Rashid

**Affiliations:** Department of Dairy Science, Bangladesh Agricultural University, Mymensingh, Bangladesh

**Keywords:** Differential enumeration, prebiotic, probiotic survivability, symbiotic

## Abstract

**Objectives:**

This study aimed to envisage the effectiveness of adding three particular prebiotics (inulin, β-glucan, and Hi-maize) to synbiotic yogurt’s physicochemical properties, sensory characteristics, and survivability of the probiotic and starter cultures.

**Materials and Methods:**

The yogurt’s gross composition, syneresis, water-holding capacity (WHC), viscosity, sensorial properties, and probiotic and starter cell stability were analyzed. The *Lactobacillus delbrueckii subsp. bulgaricus* M240-5 and *Streptococcus thermophilus* M140-2 were employed as yogurt starter bacteria, and *Lactobacillus acidophilus* LA-5 as probiotic culture. The synbiotic yogurt was formulated with 5% sucrose and 0.7% artificial vanilla flavor.

**Results:**

The findings showed that when prebiotic ingredients were added to synbiotic yogurt, it had a significant impact on its sensory qualities, WHC, syneresis, and viscosity when compared to plain yogurt samples. The prebiotics did not affect the pH and titratable acidity of the yogurt samples. Additionally, the prebiotic supplementation did not influence the protein and fat content of synbiotic yogurt (*p *< 0.05). Prebiotics had an impact on the probiotic cell viability and total viable count (*p *< 0.05) compared to the plain sample, the 2.5% β-glucan, 1.5% and 2.5% Hi-maize samples had the highest mean viability (8.95 Log CFU/ml). The starter culture ratio remained stable in response to the prebiotic levels.

**Conclusion:**

In summary, the production of synbiotic yogurts supplemented with Hi-maize and β-glucan at 1.5% and 2.5%, respectively, is highly advised because these supplementations provide yogurt with acceptable syneresis, viscosity, WHC, and sensory attributes.

## Introduction

Yogurt is a semi-solid fermented milk product made by the action of particular lactic acid bacteria (LAB;*Streptococcus thermophilus *and* Lactobacillus delbrueckii *subsp.* bulgaricus*) when incorporated with warmed milk [1]. Standard culture yogurt and bioyogurt, often known as probiotic yogurt, are the two main types of yogurt [2]. Typically, the starter culture strains used to make standard yogurt include *L. delbrueckii *subsp.* bulgaricus *and* S. thermophilus *[1]. At the same time, probiotic bacteria that are said to provide a variety of health advantages are added to probiotic yogurt and should be present in sufficient numbers [3]. The two most consumed foods worldwide for probiotic bacteria supplementation in the human diet are yogurt and fermented milk. A growing number of studies have found that regularly drinking fermented milk and yogurt has health benefits [4]. The popularity of a new range of dairy products, including synbiotic yogurt containing both probiotics and prebiotics, has been rising [5]. In dairy products, probiotic LAB have been found to produce large amounts of bactericidal proteins [6]. Consequently, synbiotic yogurt has gained popularity as a functional food that enhances human health [7].

Functional foods with the potential to treat or prevent gastrointestinal and cardiovascular disorders, as well as cancer, have been pushed to be developed as a result of changing eating patterns and dietary trends. Probiotics and prebiotics have been added to food, contributing to many of these advancements. Probiotics are live microorganisms that give consumers health advantages when ingested in sufficient proportions [10^6^–10^7^ colony-forming units (CFU)/gm or ml] [8]. Prebiotics are non-digestible food components that help form beneficial intestinal bacteria [9]. Prebiotics must be consumed in portions of 2.5 gm daily at a specific concentration [10]. They include inulin, fructo-oligosaccharides, galacto-oligosaccharides, among others [9]. Few studies have looked at the survivability of probiotic bacteria in fermented dairy products, especially yogurt. Few studies have investigated the effects of prebiotics on the sensory and textural aspects of probiotic yogurt. Compared with plain yogurt, some studies found better flavor and texture attributes of probiotic yogurt containing prebiotic compounds [11,12]. However, comprehensive research on the influences of adding commercially well-known prebiotics (inulin, Hi-maize, and β-glucan) on the physicochemical, sensory, syneresis, and water-holding characteristics of synbiotic yogurt is scanty and requires enrichment.

On the other hand, ensuring the functionality of foods is one of the most difficult aspects of incorporating probiotics and prebiotics in fermented dairy products. Several factors, such as interaction with the combined starter culture, low proteolytic activity, post-acidification, exposure to oxygen, and low temperatures during storage, harm the stability of the added microbiota in yogurt. This makes the products ineffective in providing health benefits to consumers [4]. If the prebiotic components are added to the yogurt, it promotes synergies among different components of the product, and subsequently, many alterations have occurred in the sensory attributes of yogurt [13]. Furthermore, it may influence the way products ferment and the development of LAB [14]. Therefore, we hypothesized that the association between the starter probiotic strain and prebiotic ingredients might give yogurt a better textural characteristic, microbial profile, and sensorial properties that are directly correlated with the mouthfeel and consumer preferences of these products. Considering all the factors mentioned above, this study was designed to see the changes in gross nutrient composition, physicochemical attributes, sensorial characteristics, and starter and probiotic cell viability in synbiotic yogurt.

## Materials and Methods

### Starter and probiotic cultures

The starter cultures of *L. delbrueckii *subsp.* bulgaricus *M240-5 and *S. thermophilus *M140-2 [15] were collected from the Departmental Dairy Microbiology and Biotechnology Lab at Bangladesh Agricultural University, and the probiotic culture of *Lactobacillus acidophilus* LA-5 was collected from CHR Hansen Pty Ltd. (Baswater, VIC, Australia). In starter culture propagation, De Man Rogosa and Sharpe’s broth (MRS, Merck, India) were used. The bacterial strains M240-5, M140-2, and LA-5, contained 7.5, 7.2, and 8.1 Log10 CFU/gm of freeze-dried culture, respectively. Then, freshly prepared cultures (1%) of each strain were incubated at 37°C for 24 h in MRS broth (20 ml). Centrifugation at 6,000 × g for 10 min was performed to remove bacterial cells and hold in 10 ml of 12% sterile reconstituted skim milk (Himedia, India) supplemented with 2% glucose and 1% yeast extract. These cell suspensions were stored at 4°C for further use. Before the production of yogurt, the working culture was successfully propagated 3 times.

### Manufacture of synbiotic yogurt

The vanilla flavor was chosen because it is the most commonly used flavor in yogurt and is prevalent worldwide. Sucrose concentration and artificial vanilla flavor were used at 5% and 0.7%, respectively [16]. The processing of synbiotic yogurt was carried out following the method of Pinto et al. [17], with slight modifications. Briefly, whole milk containing 3.5% fat and 8.5% solid not fat was heated at 85°C for 30 min, and during heating, 5% sucrose and 0.7% vanilla flavor were added. Afterward, milk was cooled to 42°C to add freeze-dried cultures of *L. delbrueckii *subsp.* bulgaricus*, *S. thermophilus*, and the probiotic strain *L. acidophilus* at 100 mg of each per liter. Inulin, β-glucan, and Hi-maize (IIDEA, Jalisco, Mexico) were added at two levels (1.5 and 2.5 gm per 100 gm of yogurt), and the control yogurt had no added prebiotics. All these seven (three prebiotics with two different concentrations plus a control) types of yogurt were incubated for fermentation at 37°C for 4 h (pH 4.5). The yogurts were stored in a refrigerator (1°C–5°C), awaiting subsequent analysis.

### Physicochemical measurements

The physicochemical analysis was performed to estimate the titratable acidity, pH, total solids, protein, fat, fiber, ash, water holding capacity (WHC), syneresis of yogurt, and apparent viscosity. The Babcock technique was used for fat percentage estimation; titratable acidity was estimated by titrating the sample with 0.1 N NaOH solution, while phenolphthalein was used as an indicator. Total solids were measured at 105°C with overnight drying of the yogurt sample to constant weight using a hot air oven (Thermoline Scientific, Australia). The ash content was determined using an electric muffle furnace with a solid material explosion at 550°C (Labec, Australia). A modern pH meter (Hanna, Romania) was applied to record the pH of yogurt using well-mixed yogurt samples. The Kjeldahl method was used to measure the protein content. As per the details described by James [18], the total solids, ash, fat, and protein contents were assayed. The fiber content was assessed by the enzymatic-gravimetric method [19]. In brief, the samples were gelatinized using heat-stable -amylase (Himedia, India). After gelatinization, the samples were digested using amyloglucosidase and protease (Sigma-Aldrich, Germany). Successively, distilled water (60°C) was used to filter and wash the fiber. Afterward, the residues were dried overnight at 105°C in an oven and weighed.

The viscosity of the yogurt samples was measured at 25°C using a viscometer (Brookfield). The spindle number used was LV 2, which ran at 0.5 rpm. At the end of the 15th sec of the assessment period, the readings were noted as centipoises (cP). According to the procedure outlined by Isanga and Zhang [20], yogurt’s susceptibility to syneresis (STS) was assessed. In short, 100 gm of yogurt sample was placed in a funnel lined with a Whatman filter paper (no. 1). The amount of whey stored in a beaker was measured, and the STS was estimated using the following formula after draining for 6 h at room temperature.

STS (%) = V1/V2 *×* 100

Where, V1 = Volume of whey collected after drainage; V2 = Volume of yogurt sample.

The WHC of yogurt was measured according to the method of Isanga and Zhang [20]. Five grams of yogurt was centrifuged at 4,500 *×* g for 30 min at 10°C. The WHC was calculated using the formula below.:

WHC (%) = (1-W1/W2) *×* 100

Where, W1 = Weight of whey after centrifugation, W2 = Yoghurt weight. 

### Microbiological analysis

To determine the total viable count (TVC), the method was followed as described by Islam et al. [21]. In short, 1 ml of the sample was mixed with 9 ml of diluent (0.85% NaCl solution), and the mixture was vortexed thoroughly. Then, 10^−1^ to 10^−9^ serial dilutions were prepared. As every dilution increased from 10^−5^ to 10^−9^, 0.1 ml of the test sample was inoculated at 37°C for 24 h onto a plate count agar (Himedia, India). Plates containing 30 and 300 colonies were counted after successive incubations. The number of CFU was measured by the dilution factor and the plated sample volume and finally converted into Log CFU per ml yogurt sample. 

Yogurt samples containing *S. thermophilus *wereincubated aerobically at 37°C for 72 h and enumerated using M17 agar (Merck, Germany). In contrast, *L. acidophilus* from the yogurt samples was enumerated using 5-bromo- 4-chloro-3-indolyl-β-d-glucopyranoside (X-Glu) (Merck, Germany) added into Rogosa Agar (Merck, Germany) at a concentration of 40 μg/ml. This revealing method is based on a particular 1 chromogenic reaction with X-Glu to visualize the D-glucosidase activity of a particular strain of *L. acidophilus *[22]. Inoculated X-Gluagar plates were incubated anaerobically for 72 h at 37°C. A dark blue colonyformation indicated plates with* L. acidophilus*, while plates containing* L. bulgaricus* formed white zones*.*

In addition, coliform, yeast, and mold counts were also performed according to Marshal [23]. In the case of counting yeast and mold, potato dextrose agar and tartaric acid were used. Enumeration was performed through the pour plate technique, incubated at 30°C for 3–5 days. The most probable number method with violet-red bile agar (Himedia, India) was applied for coliform counting.

### Sensory analysis

A panel of 30 people (15 males and 15 females) between 20 and 50 years old comprised the panel to assess the sensory profile of yogurts. The students and faculty members of Bangladesh Agricultural University comprised the tasting panelists. Each participant received seven yogurt samples to taste, assess, and comment on the sensory qualities of each serving. The 9-point hedonic scale means: like extremely = 9, like very much = 8, like moderately = 7, like slightly = 6, neither like nor dislike = 5, dislike slightly = 4, dislike moderately = 3, dislike very much = 2, dislike extremely = 1. 

### Statistical analysis

There were three trials for product manufacturing, and all the laboratory analyses were done in triplicate. One-way source of variation analysis was done using Minitab version 17. Tukey’s test was employed for multiple comparisons whenever needed.

## Results and Discussion

### Physicochemical analysis

The findings of the physicochemical analyses are presented in Table 1. The prebiotics did not affect the pH or titratable acidity of the yogurt samples. The pH ranged from 4.31 for synbiotic yogurts with prebiotics to 4.43 for control samples for each kind of yogurt (Table 1). The pH levels of the synbiotic yogurts were lower than those of the persimmon fruit-based yogurts in Arslan and Bayrakci [24] report, which may be because different amounts of prebiotics were used to produce the yogurt. According to Çakmakçi et al. [25], the pH of yogurt containing banana marmalade varies between 4.07 and 4.60 during storage. These findings are consistent with the results of our investigation. The titratable acidity of synbiotic yogurt ranged from 1.05% to 1.17%, where the maximum titratable acidity (1.15%) was observed on the effect of Hi-maize (2.5%), and the lowest titratable acidity (1.05%) was observed on the effect of β-glucan (1.5%) (Table 1). The ongoing metabolic activity of probiotics or starter cultures in the food system may explain this decline. Others have previously reported similar outcomes in the case of acidity [26]. Furthermore, Kumar and Kumar [27] found that yogurts containing *L. rhamnosus* and different fruits had an average titratable acidity that ranged from 0.45% to 0.71%; nonetheless, our results were higher than those previously reported.

### Yogurt composition

Synbiotic yogurts’ composition (%) is presented in Table 1. From the findings of our study, total solids (17.15–17.91), protein (4.52–4.6), fat (5.71–5.79), ash (0.91–1.05), and fiber (0–1.55) are in line with the previous studies [10]. No influence of added prebiotics was found on the protein and fat content of synbiotic yogurt (*p* > 0.05), although the addition of inulin, β-glucan, and Hi-maize at rates of 1.5% and 2.5% resulted in lower ash content in yogurt when compared to the plain samples (*p* < 0.05). In the case of fiber content, all prebiotics are intended to improve the fiber percentage (*p *≤ 0.05) once they consist of soluble fibers. The fiber content of the control sample was 0.00%, and the fiber content of yogurts after adding prebiotics was 0.83% to 1.55%. In addition, some of the components may have been lost during the whey syneresis, and the analysis of these components using the dietary fiber technique may result in under-quantifications [13].

### Syneresis, WHC, and viscosity

Syneresis results from liquid oozing out of the yogurt curds, an unpleasant quality in yogurt products [28], primarily due to its increased total solids, protein, fat, and ash content. Yogurt with a high-fat content has been linked to decreased syneresis attributes [20]. In this study, the addition of various prebiotic levels had a significant impact on the syneresis of synbiotic yogurt. Despite the flavors and prebiotics that have been added, plain yogurt was found to have much lower syneresis than yogurts that have been supplemented (Table 1). Syneresis of plain yogurt was 19.72%, whereas, after the addition of different levels of prebiotics, it became within the range of 21.03% to 25.88%. It is indicated that the addition of inulin and β-glucan increased syneresis compared to Hi-maize. Notably, there was no discernible change in the treatments of the same prebiotic at 1.5% and 2.5%, except for samples containing β-glucan, inulin, and Hi-maize. The observed behavior of yogurt was in line with the results reported by Vasiljevic et al. [29], who observed the effect of adding β-glucan and inulin on the syneresis effect of probiotic yogurt and revealed that the incorporation of prebiotics enhanced the yogurt’s syneresis. They attributed the observed behavior to the presence of long-chain polysaccharides, which may interact with casein to produce a three-dimensional structure, forming a weaker gel with less capacity to hold water. In this study, except Hi-maize (1.5% and 2.5%), all of the prebiotic-containing treatments in the current investigation had more syneresis than the control. Heydari et al. [12] observed that β-glucan addition did not impact the WHC of non-fat yogurt, which is related to the findings of the current investigation. However, according to Guven et al. [30], adding inulin to yogurt had no impact on syneresis.

In the present investigation, plain yogurts resulted in lower WHC (60.14%) compared to synbiotic yogurts with different levels of prebiotics ranging from 62.00% to 65.27%, where higher WHC (65.27%) was found in the yogurt with 2.5% Hi-maize (Table 1). It could be due to the higher protein and fat content, as well as the effects of adding probiotics to synbiotic yogurt versus plain yogurt. According to Cortez-Trejo et al. [28], the WHC of yogurt is proportional to the proteins’ capability to hold water. Milk fat globules also play a big role in keeping the water in the yogurt structure, as suggested further. 

When compared to all the prebiotics added to synbiotic yogurts, plain yogurt’s viscosity was found to be lower, which is consistent with the higher level of total solids in synbiotic yogurts as described by Isanga and Zhang [20] and Marshall [23]. According to some studies [20], a higher viscosity can be achieved if the yogurt has a higher fat content. In this study, the viscosity of plain yogurt was 13,834.00 cP; after adding prebiotics of different levels, the viscosity of yogurt increased to 21,834.62–23,925.42 cP (Table 1).

### Sensory properties

**Figure 1.**
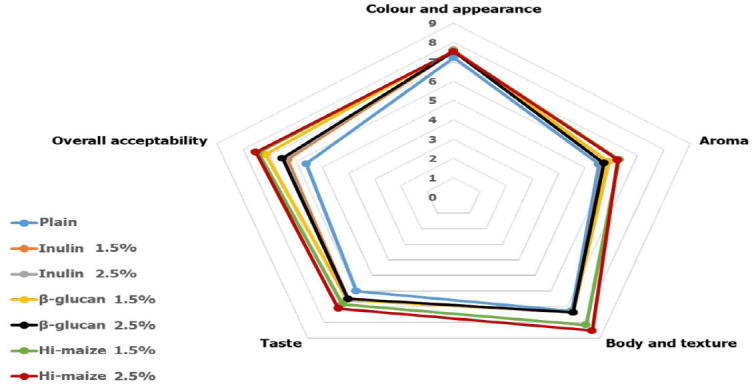
Sensorial attributes of symbiotic yogurts compared with plain samples and scored by using a 9-point hedonic scales resulting from a tasting panelist (*n* = 30).

Figure 1 shows the results of the sensory analysis of the yogurt samples. The incorporation of different levels of prebiotics had no significant effects on color, appearance, or aroma. The supplementation of prebiotics influenced the body and texture of yogurt, taste, and overall acceptability. In 1.5% and 2.5% Hi-maize yogurt, body, texture, and taste scored the highest, followed by β-glucan and inulin, while plain samples scored the lowest. The acceptability of synbiotic yogurt was increased after adding prebiotics at different concentrations compared to plain yogurt. The overall acceptability of plain yogurt was 5.61, and the prebiotics-fortified samples’ scores ranged from 6.32 to 7.53, whereas 1.5% and 2.5% Hi-maize and 1.5% β-glucan yogurt were recorded with the highest overall acceptability. However, studies on the impact of various prebiotics on the sensory characteristics of fermented milk products have shown conflicting results. Sarwar et al. [31] added inulin to yogurt samples, resulting in good flavor and texture properties compared to plain samples. In a study, Hussien et al. [32] claimed that fat-free yogurt with inulin and *L. acidophilus* had a pleasant flavor. In another study, Heydari et al. [12] revealed the conflicting results of adding β-glucan to yogurt. In a report by Helal et al. [33], it was evident that inulin inclusion decreased the organoleptic attributes of yogurt, which supports the findings of our study. However, Staffolo et al. [34] observed no appreciable variations in overall acceptance between yogurts containing 1.3% inulin and those containing none at all.

**Table 1.** Inulin, β-glucan, and Hi-maize effects on the physico-chemical characteristics of synbiotic yogurts at 1 week after production (*n* = 3).

Characteristics	Plain	Inulin levels		β-glucan levels		Hi-maize levels		*p*-value
		1.5%	2.5%	1.5%	2.5%	1.5%	2.5%	
Titratable acidity (%)	1.17 ± 0.02	1.14 ± 0.03	1.11 ± 0.02	1.05 ± 0.01	1.13 ± 0.01	1.12 ± 0.03	1.15 ± 0.04	0.211
pH	4.43 ± 0.21	4.33 ± 0.10	4.32 ± 0.12	4.36 ± 0.10	4.31 ± 0.20	4.37 ± 0.20	4.41 ± 0.20	0.345
Total solids (%)	17.15^b^ ± 1.40	17.29^b^ ± 1.32	17.65^a^ ± 0.77	17.25^b^ ± 0.62	17.69^a^ ± 1.00	17.78^a^ ± 0.98	17.91^a^ ± 0.56	0.041
Protein (%)	4.55 ± 0.75	4.54 ± 0.72	4.52 ± 0.21	4.53 ± 0.31	4.59 ± 0.25	4.59 ± 0.31	4.60 ± 0.45	0.154
Fat (%)	5.72 ± 0.22	5.74 ± 0.32	5.71 ± 0.12	5.70 ± 0.17	5.73 ± 0.18	5.76 ± 0.19	5.79 ± 0.15	0.214
Fiber (%)	0.00	0.83^c^ ± 0.01	0.95^c^ ± 0.02	1.25^b^ ± 0.01	1.54^a^ ± 0.05	1.32^b^ ± 0.02	1.55^a^ ± 0.02	0.031
Ash (%)	1.05^a^ ± 0.04	0.92^b^ ± 0.05	0.93^b^ ± 0.02	0.91^b^ ± 0.02	0.92^b^ ± 0.03	0.93^b^ ± 0.02	0.94^b^ ± 0.03	0.010
STS (%)	19.72^c^ ± 0.35	24.19^a^ ± 0.21	25.88^a^ ± 0.44	25.88^a^ ± 0.15	24.19^a^ ± 0.11	21.11^b^ ± 0.31	21.03^b^ ± 0.37	0.021
WHC (%)	60.14^c^ ± 0.53	62.00^b^ ± 0.52	62.52^b^ ± 0.18	64.52^a^ ± 0.32	64.00^a^ ± 0.97	64.11^a^ ± 0.43	65.27^a^ ± 0.50	0.033
Viscosity (cP)	13,834.00^c^ ± 204.00	21,834.62^b^ ± 50.23	22,456.28^b^ ± 43.27	237,689.21^a^ ± 65.45	23,925.42^a^ ± 38.92	23,423.28^a^ ± 70.04	23,264.29^a^ ± 64.39	0.016

STS, Susceptibility to syneresis; WHC, Water holding capacity; cP, Centi poise.

^abc^Mean values in a row with uncommon superscript letter differed significantly.

### Microbiological characteristics

The data regarding microbiological analysis are shown in Table 2. Prebiotics greatly influenced the cell viability (*p *= 0.032), where the greatest viability was observed in 2.5% β-glucan, 1.5% and 2.5% Hi-maize compared to the plain yogurt. The starter culture counts for* L. bulgaricus *(7.45–7.85 Log CFU/gm) and* S. thermophilus* (7.21–7.79 Log CFU/gm) remained stable in response to the prebiotic levels. However, Streptococci remained static in the starter culture ratio compared to the Bacilli group. Significant differences existed in the overall viable count (*p *= 0.041) among the samples. In contrast, the highest values were recorded in 2.5% added inulin (8.80 Log CFU/gm), ­β-­glucan (8.97 Log CFU/gm), and Hi-maize (8.96 Log CFU/gm) yogurt. In our study, the prebiotics did not affect the yeast and mold counts. Additionally, no one sample showed a coliform count. It is evidenced that the incorporation of prebiotics encourages some probiotic bacteria to proliferate [35], and the incorporation of Hi-maize increases the viability of probiotic bacteria [36]. According to Terpou et al. [37], to increase the growth and survival rate of probiotic bacteria in food products, prebiotic substances are thought to be a preferable option. Oligosaccharides, plant extracts, grain bran, lactulose, and inulin, for example, have a favorable effect on probiotic viability in yogurt during cold preservation and passage through the intestines [38]. Distinctly, the prebiotic supplement could also influence the counts of yogurt starter cultures, which may indirectly affect probiotic survivability. The probiotic microbiota and prebiotic compounds, that is, the synbiotic product, could increase the beneficial health effects of yogurt products by stimulating the multiplication and functional activities of the gut microbiota. On the other hand, fermented dairy products like yogurt and cheese undergo spoilage due to the growth of yeasts and molds. Yogurt may degrade if added fruit-like components are because they provide fermentable substrates for yeasts and molds [39]. However, a crucial factor in producing probiotic products like yogurt is the interaction between starter cultures and probiotics [40]. In this study, the number of *S. thermophilus* remained relatively static among the yogurt samples. It might be because, unlike *S. thermophiles*, high acidity has little to no impact on *L. bulgaricus*’ viability. Ranadheera et al. [41] revealed similar outcomes, with *S. thermophilus* being more stable in the yogurt matrix than *L. delbrueckii *subsp.* bulgaricus* in probiotic (*L. acidophilus* LA-5) yogurt. Additionally, it has been suggested that fermented foods carrying probiotic bacteria should meet the recommended minimum level of > 6 Log CFU/gm to optimize the optimum therapeutic potential [25]. Even though probiotic organism counts decreased during cold preservation, probiotic yogurts preserved probiotic attributes until the termination of the preservation period.

**Table 2.** The changes in microbiological characteristics of yogurt samples.

Characteristics	Plain	Inulin levels		β-glucan levels		Hi-maize levels		SEM	*p*-value
		1.5%	2.5%	1.5%	2.5%	1.5%	2.5%		
Probiotic cell viability (log CFU/gm)	7.12^c^	7.41^c^	8.50^b^	8.51^b^	8.95^a^	8.92^a^	8.95^a^	0.712	0.032
*Lactobacillus bulgaricus* (log CFU/gm)	7.55	7.65	7.75	7.78	7.82	7.45	7.85	0.348	0.058
Streptococci (log CFU/gm)	7.21	7.51	7.66	7.73	7.77	7.72	7.79	1.275	0.063
TVC (log CFU/gm)	6.11^d^	7.43^c^	8.80^a^	8.44^b^	8.97^a^	8.41^b^	8.96^a^	0.932	0.041
Yeast and mold (log CFU/gm)	2.01	3.11	2.23	2.43	2.07	2.12	2.79	0.553	0.673
Coliform count	-	-	-	-	-	-	-	-	-

SEM = Standard error of mean; CFU = Colony forming unit.

^abcd^Mean values in a row with uncommon superscript letters differed significantly.

## Conclusion

Synbiotic yogurt is now a popular functional food all over the world. This study aimed at the influence of prebiotic incorporation on the physicochemical, sensory, and probiotic cultures’ viability. It is important to note that prebiotic addition techniques significantly improve probiotic survivability. The current investigation directed that appropriate probiotic bacteria (e.g., *L. acidophilus* LA-5) did not influence sensory acceptance or starter culture viability of synbiotic yogurt. The 1.5% and 2.5% Hi-maize and β-glucan had the best body and texture and the highest probiotic viability and dietary fiber content. Finally, it can be said that appropriate prebiotic content, suitable microbiota, and good manufacturing measures make it possible to produce synbiotic yogurt with the highest acceptance, the best health effects, and a reasonable price. However, further investigation is required into novel technologies, such as new methods and materials for dietary fiber ­supplementation, to increase probiotic microbial survival without substantially altering sensory qualities or cost.

## List of abbreviations

MRS, De Man Rogosa and Sharpe.
